# Robot-Assisted Fracture Surgery: Surgical Requirements and System Design

**DOI:** 10.1007/s10439-018-2005-y

**Published:** 2018-03-09

**Authors:** Ioannis Georgilas, Giulio Dagnino, Payam Tarassoli, Roger Atkins, Sanja Dogramadzi

**Affiliations:** 1 Bristol Robotics Laboratory, University of the West of England, Coldharbour Lane, Bristol, BS161QY UK; 2 Department of Mechanical Engineering, University of Bath, Building 4E3.45, Bath, BA27AY UK; 3 The Hamlyn Centre for Robotic Surgery, Imperial College London, London, SW72HR UK; 4 University Hospitals Bristol, Upper Maudlin Street, Bristol, BS28HW UK

**Keywords:** System design and development, Computer-assisted surgery, Medical robotics, Percutaneous fracture surgery

## Abstract

The design of medical devices is a complex and crucial process to ensure patient safety. It has been shown that improperly designed devices lead to errors and associated accidents and costs. A key element for a successful design is incorporating the views of the primary and secondary stakeholders early in the development process. They provide insights into current practice and point out specific issues with the current processes and equipment in use. This work presents how information from a user-study conducted in the early stages of the RAFS (Robot Assisted Fracture Surgery) project informed the subsequent development and testing of the system. The user needs were captured using qualitative methods and converted to operational, functional, and non-functional requirements based on the methods derived from product design and development. This work presents how the requirements inform a new workflow for intra-articular joint fracture reduction using a robotic system. It is also shown how the various elements of the system are developed to explicitly address one or more of the requirements identified, and how intermediate verification tests are conducted to ensure conformity. Finally, a validation test in the form of a cadaveric trial confirms the ability of the designed system to satisfy the aims set by the original research question and the needs of the users.

**Electronic supplementary material:**

The online version of this article (10.1007/s10439-018-2005-y) contains supplementary material, which is available to authorized users.

## Introduction

Medical devices must be well-designed to provide high quality care for patients.^28^ To be considered ‘well-designed’, a medical device must be clinically effective and safe, while also able to fulfil the needs of the users.^30^ This requires taking into consideration a number of factors including the capabilities and working pattern of the clinical users, the needs of the patients, the environment in which the device will be used, and the system(s) of which it will be part of Ref. 29. All these factors will inform the design of the device. Poorly designed devices increase the risks for human error,^23^ as well as for incidents and accidents in medical care.^5^

To increase the adoption rate of a medical device, developers must have a clear and thorough understanding of the clinicians, patients and carers who will use the device.^30^ Conducting an early user research is necessary for developers to understand and specify the context of use and the user and organizational requirements.^24^ Failing to adequately study the potential users at the beginning of development may result in assumptions about their needs, capabilities and characteristics. So, the device will be developed and evaluated based on incorrect information. This has serious implications not just for the safety of the new device, but also for its commercial success.^30^ The development of medical devices in both commercial and research domains^1^ as well as the regulatory bodies, i.e. Food and Drug Administration (FDA) in the US and Medicines and Healthcare products Regulatory Agency (MHRA) in the UK, strongly suggest that a user-driven approach is necessary to ensure a functional product for the clinical, safety–critical environment.^34^

Although some manufacturers of medical equipment already integrate human factors principles in their products, there is still a lack of commensurate work on the practicalities of such engagement.^6^ Therefore, a user-centred approach should be conducted at the early stage of a development project in order to obtain a better and safer product^7^ by including the needs and views of the users.

Based on the user needs, a set of requirements can be developed to drive the design process. Unfortunately, the needs are usually abstract and expressed in natural language making it difficult to formulate technical specifications. Capturing and organising requirements is a crucial part of the design process.^16^ Technical requirements can be derived by using user proxies in the form of expert evaluators.^35^ A framework using ontological charts to capture the user needs along with other constrains and assist with the design process for medical devices has been proposed.^21^ A common theme in incorporating user-views is using information modelling techniques,^22^ for example the V-model of design.^18^

The V-model is a waterfall approach that encourages up-front planning for the development process. It allows for a systematic testing and validation regime for the entire development life-cycle,^31^ aiming to follow a good design approach that incorporates validation as a main development activity and not an afterthought.^2^

In this paper we describe the user-driven approach used in designing and developing a system for robot-assisted fracture surgery (RAFS). The RAFS project aims to develop a robotic system that assists surgeons to perform reduction of intra-articular fractures in a minimally invasive way. It provides the surgeon with physical and virtual assistance to minimise operational time and issues associated with open surgery (i.e. infection), leading to shorter recovery times and post-operation costs. The system has been developed in close collaboration with clinicians and has been tested in realistic conditions.^13^

**Figure 1:**
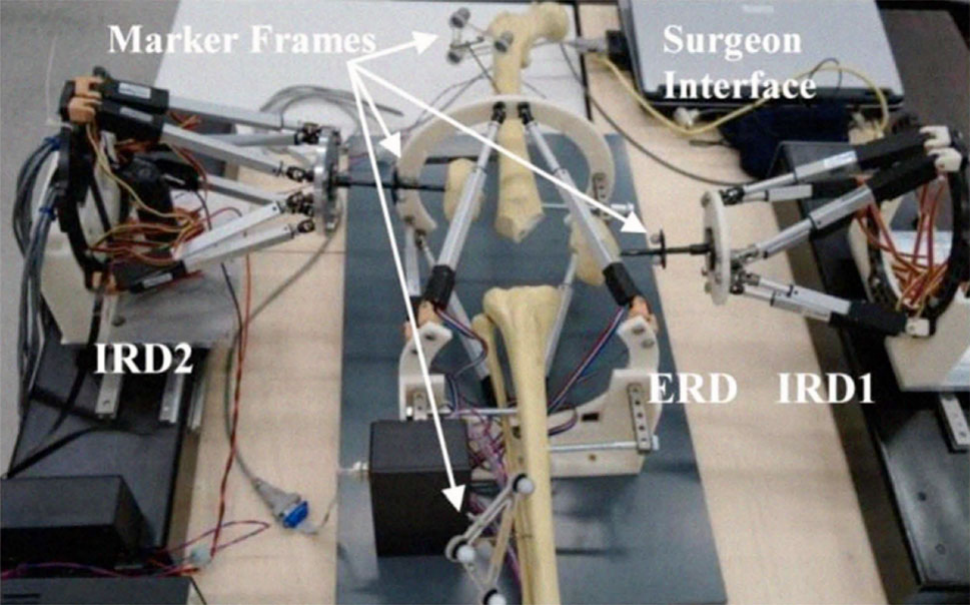
Initial prototype of Robot-Assisted Fracture Surgery system for minimally invasive reduction of distal femur fractures developed in the Bristol Robotics Laboratory (BRL). The system comprises of one parallel robot for manipulating the tibia bone (ERD) and two parallel robots for manipulating the medial and lateral condyle fragments (IRD1, IRD2), a motion controller, a marker based navigation system and the surgeon interface.

User-driven design is widely implemented in robotic applications for medical systems.^25^,^26^ The approach proposed here is based on an early-stage user study, to capture user needs, and the V-model of system development. The user study consists of a series of interviews with surgeons to understand the clinical practice, instruments used, and procedural challenges. An earlier prototype of RAFS (Fig. 1)^33^ was presented to provide context for the interviews. Based on the information gathered the requirements for the system were elicitated and using the V-model the system was developed by a suitable workflow, system architecture and sub-systems along with their respective testing criteria and metrics. The individual functionality has been verified at the sub-system level and integrated and tested to the complete system. The final testing and validation was conducted on cadaveric specimens demonstrating the ability of the re-designed system to satisfy the originally set requirements. A final user study was conducted after the system validation to gather clinicians’ assessment of the test results and potential utilization of the system in the clinical practice. This was part of a broader health economics and market research of robot-assisted fracture surgery.

**Figure 2:**
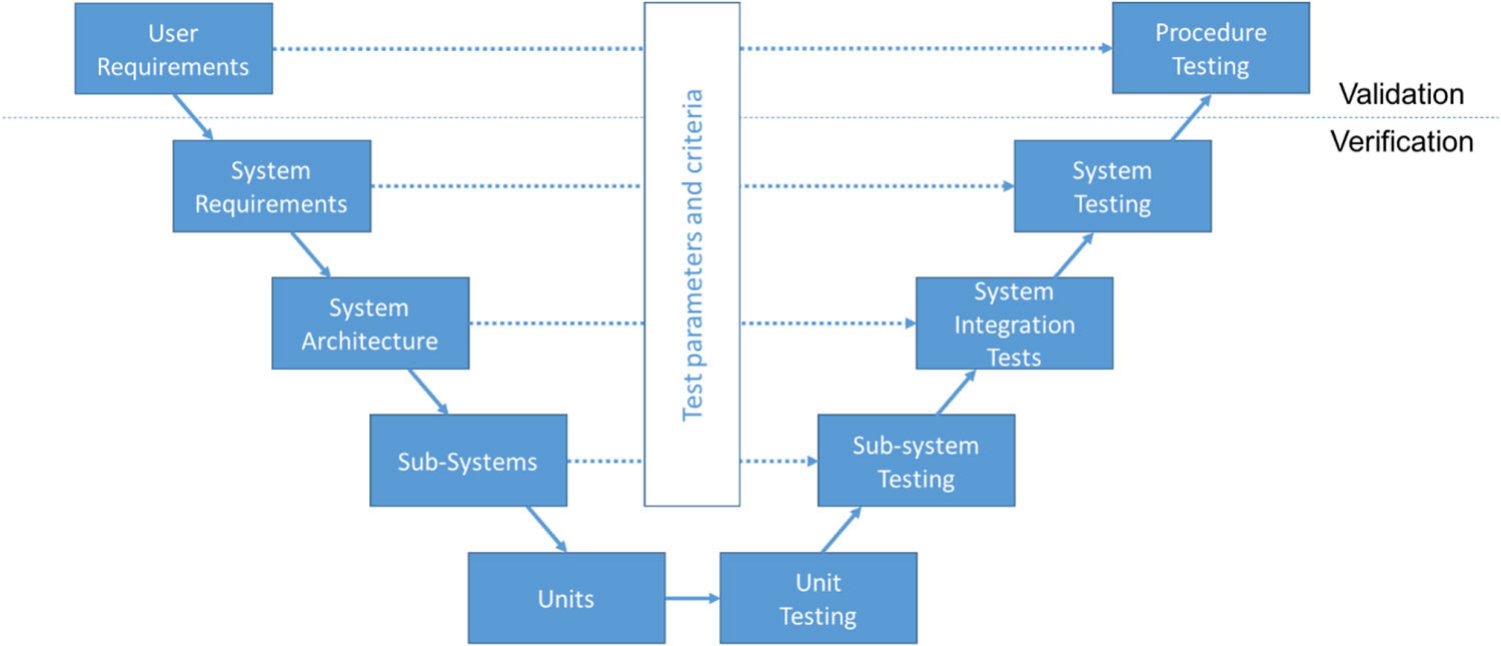
The V-model of design that is used in the development of the RAFS system. On the left side is the progressively increased resolution of the technical specifications while moving downwards the systems. On the right side is the integration and testing steps towards the full system. The horizontal arrows indicate that part of each step is the establishment of criteria and parameters to be used in the testing phase to evaluate the success of an integration step. The top level actions (user requirements and final testing) are the validation steps of the development while the rest are the verification steps for the different elements of the system.

This paper will present the methods used, namely the qualitative method and the details of end-user interviews and the V-model of design for system development. In the results section we will first present the requirements that have been derived from the interviews and then how these have been met by the architecture, workflow, and sub-systems of the RAFS system. Finally, at the discussion we will summarise lessons learned from the design process.

## Materials and Methods

### Qualitative Methods

The end-user part of the design process involved a qualitative study conducted through interviews with orthopaedic surgeons experienced in intra-articular fracture reduction. This study consisted of two phases: (1) define the objectives of the study; and (2) conduct interviews with potential clinical users of the device to specify the requirements for the RAFS system.

#### Research Objectives

The RAFS development team (DT) is composed of three engineers and two orthopaedic surgeons. In the first phase of this study, the DT discussed identification of potential users and applications for the proposed device in order to focus the study on the needs of the users and to collect data that can be easily implemented in the design and development process.

The DT recognised the following research objectives:

- Identify the target clinical users.
- Identify the potential clinical application for the system.
- Identify barriers to safe and effective system design/development/adoption.
- Collect user opinions on possible design features.
- Refine and validate the concept for the new device.

The DT identified that the most suitable pilot clinical application for RAFS is a distal femoral fracture (DFF). This was due to the large fragments created in this kind of trauma and the relatively simple soft tissue structures in the region of the distal femur when approaching from the anterior side. For this reason, potential users for the RAFS device are orthopaedic surgeons with expertise in knee fracture reduction.

#### Interviews with Clinical Users

In the initial user study a total of 13 individual face-to-face, semi-structured interviews were conducted with experienced (average experience ≈ 16 years) orthopaedic surgeons from the UK, and the EU (Table 1). Each interview lasted between 25 and 60 min. During the interviews we adopted an approach for the surgeons to discuss as freely as possible joint fracture reduction surgery and related issues and limitations. Probing questions were used when necessary to encourage the surgeons to provide more details. Additional questions were used to clarify the themes of major interest.

**Table 1:** Clinical users: orthopaedic surgeons interviewed.

Gender	Clinical role	Experience (years)	Region
Male	Consultant	14	UK
Female	Registrar	8	UK
Male	Consultant	22	UK
Male	Consultant	22	UK
Male	Registrar	8	UK
Male	Consultant	25	UK
Male	Registrar	9	UK
Male	Consultant	10	UK
Male	Consultant	16	UK
Male	Consultant	7	EU
Male	Consultant	8	UK
Male	Consultant	30	UK
Male	Professor	28	EU

The aims of the interviews were: (1) to investigate the current state-of-the-art in joint fracture reduction surgery with focus on DFFs; (2) to investigate limitations and issues related to the current surgical procedure; (3) to define users’ requirements for RAFS in terms of its operational characteristics (e.g. size, integration in Operating Theatre (OR), interaction, *etc*.); 4) to define expected medical functions for RAFS. Familiarity with other robotic systems and image-based technologies was taken into account to normalise the sample for personal experiences and preferences.

A broader market research was conducted by an external company. As part of that 18 Orthopaedic Surgeons and Heads of Orthopaedic Departments from US, UK, and Germany were interviewed. The aim of these interviews were to assess the potential of (1) the system adoption from the financial viewpoint, (2) the proposed clinical workflow, and (3) the usability of RAFS. The results related to (2) and (3) will be further discussed.

#### Data Analysis

The recordings were transcribed for the data analysis to produce results strictly linked with the research objectives defined by the DT in the first phase of this study.

The interviews were transcribed, categorised and coded according to the grounded theory method.^4^ Categories and example codes are showed in Table 2.

**Table 2:** Data analysis: categories and coding.

Categories	Current JFR procedure description	Current related issues	Clinical needs	Expected medical functions for RAFS	Expected benefits	Barriers
Codes	Open surgery	Visualization	Pre-operative imaging	Size/weight	Intra-operative imaging	Osteoporotic bones
	Minimally invasive surgery (MIS)	Access	Intra-operative imaging	Speed	Fracture reduction accuracy	Soft tissues management
	Surgical workflow	Reduction accuracy	Soft tissue damage	Portability	Soft tissues preservation	Complex fractures (# fragments)
	Imaging	Soft tissues damage	Reduction accuracy	Reduction accuracy	Earlier surgery	Time
	Fracture reduction	Osteoporosis	Manual dexterity	Soft tissues management	Patient outcome	Integration in OR
	Fracture fixation	Reduction evaluation		Imaging	Faster rehabilitation	Integration with surgeons
	Workspace			System control	Arthritis avoidance	Fracture fixation
	Soft tissues management			GUI	Reduced hospitalization time	Sterilization
	Osteoporotic bones			Safety	Reduced NHS costs	Costs
	Outcome evaluation			Sterilization		
	Heling time			Integration		
				Proof of concept		
				Other fractures		

The coded data revealed surgeons’ ideas and opinions (common and conflicting) from which we generated the system requirements.

### Operational, Functional, and Non-Functional Requirements

There are different types of requirements. The operational requirements define the major purpose of the system. Functional requirements specify what the system has to do in order to satisfy the operational requirement. Non-functional requirements define system constrains or modifying influences on the system. Non-functional requirements can be split into the performance requirements that define how a function should be implemented and system requirements on external parameters that are affecting the design of the system. The non-functional requirements can lead to errors and safety compromises^15^ and should be defined using methods to ensure appropriate definition.^20^,^27^

In this work, an approach similar to the work from Ulrich and Eppinger^17^ is followed. Namely, the user defines the operational requirement, and in the case of an expert user, provides insights into functional and non-functional requirements. The requirements are organised in a hierarchy, with functional requirements being the top-level requirements and the non-functional requirements being more detailed. Most requirements will be defined from regulatory, safety, and environment constrains that can be initiated by a user but involve a degree of expanding based on the technical literature and practice. In the specific study, the regulatory and safety environment was dictated by current FDA and MHRA guidance and requirements.^32^ Based on this analysis, the coded data was converted into functional and non-functional requirements. The main approach was to convert any need or desire expressed from the users into a technically described description. For functional requirements the system was required to “achieve” a goal, while for non-functional requirements the system was required to “satisfy” a criterion.

### V-model of the Development Process

The V-model is based on the principle that the development process is moving from the generic to the specific up to the lowest level of resolution usually the component level and then the integration process follows the reverse direction. It is important to note that the downwards process is not only setting requirements and technical specifications but also the criteria and methods for testing integration on the upwards direction (2).

The implementation of the V-model needs a description of the overall system architecture in order to satisfy the criteria, i.e. the fundamental components required to achieve the functional requirements. Based on these division of functionality, each sub-system is described in detail to address the requirement. Finally, the units of the sub-systems are defined to address technical functions.

## Results

### User Study Outcomes

**Figure 3:**
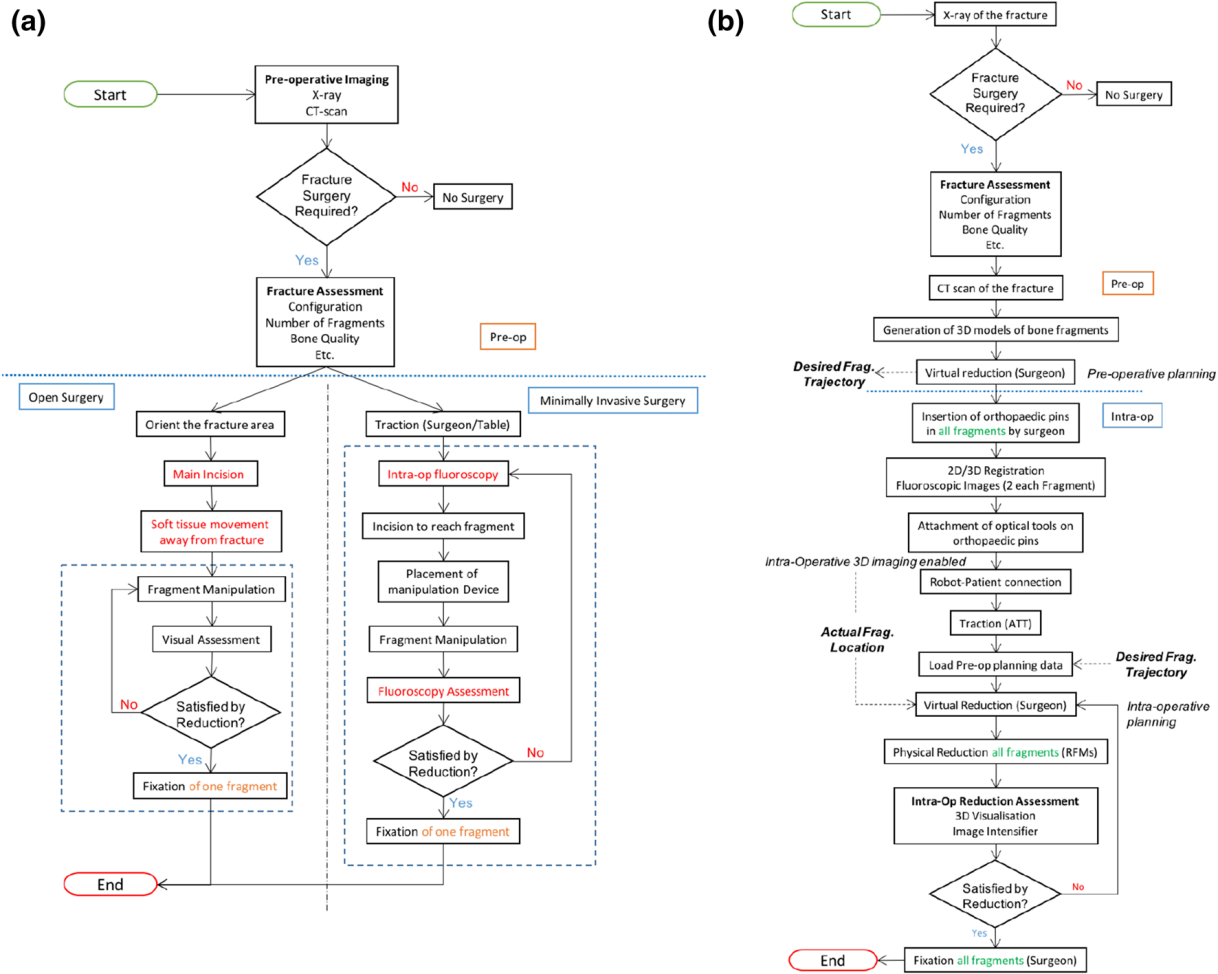
Workflow for distal femur fracture surgery. (a) is the workflow currently for open-surgery and minimally invasive surgery for DFF as described from the user-study; (b) is the workflow as has been developed based on the requirements and the use of the RAFS system.^33^

One key point in the development of a new medical device is to understand the application field of the system. The results from the qualitative study emphasised the current surgical procedure and the limitations for using a minimally invasive approach in DFF surgical management. A summary of the state-of-the-art in surgical treatment of DFFs, from the diagnosis of the fracture to the post-operative evaluation of the patient’s outcome is presented in Fig. 3a.

The investigation of the minimally invasive surgical management of DFFs highlighted the limitations related to the current procedure. Four key issues emerged from the interviews with the orthopaedic surgeons with a prominent level of consensus: (1) poor surgical site imaging; (2) difficult access through small incisions; (3) challenging and often inaccurate reduction of bone fragments followed by disadvantages of the external fixation; and 4)soft tissue damage due to the lack of the site visualisation.

### Requirements Generation

Based on the interview results, the requirements that the RAFS system should address can be summarized into operational, functional and non-functional. The hierarchy of requirements is as follows: the operational requirement at the top, functional requirements at the component level and Non-Functional Requirements in the third tier providing a context for the Functional Requirements. The hierarchy of Functional and Non-Functional requirements are summarized in Table 3.

**Table 3:** Requirements and description.

Requirement number	Description
FR1	The system can access the IJF from different positions
FR2	The system can attach to IJF fragments
NFR1	The system deals with both normal and osteoporotic bones
NFR2	The system is able to deal with the soft tissues around the fracture minimizing the “biological cost” of a big incision
FR3	The system manipulates IJF fragments (i.e. rotation and translation)
NFR3	The system creates sufficient working space inside the joint
NFR4	The system allows the surgeon to perform fracture fixation
FR4	The surgeon is in control of the operation of the system
NFR5	The system is under the surgeon’s continuous supervision
NFR6	The system has an intuitive graphical user interface (GUI)
NFR7	The system is user-friendly
FR5	The system enables visualization of IJFs
NFR8	The system creates a 3D models of the fracture;
NFR9	The system visualises the 3D models of the fracture
NFR10	The system allows pre-operative planning of the JFR
NFR11	The system tracks in real-time the actual position of the fracture and updates the position of the 3D models
Size considerations	
NFR12	The system adapts to any standard operating room
NFR13	The system is portable
NFR14	The system allows the use of image intensifier in operating room
NFR15	The surgeon has access to the surgical field
Safety considerations	
NFR16	The system conforms to the regulations in force
NFR17	The system is not traumatic for the patient

*Operational requirements* A system that will enable and assist surgeons in the performance of reduction of intra-articulate joint fractures (IJF) in a minimally invasive manner within existing clinical practice and national health system protocols.

The *functional requirements* (FRx) have also been as follows

1. The system can access the IJF from different orientations (i.e. different angles)
2. The system can attach to IJF fragments
3. The system manipulates IJF fragments (i.e. rotation and translation)
4. The surgeon stays in control of the system operation
5. The system enables visualization of IJFs

From interviews some of the *system non-functional requirements* have been defined but further ones were created to comply with safety and certification procedures for medical devices.

As an added safety criterion, a study to collect force data in fracture reduction orthopaedic operations^9^,^19^ were conducted providing specific thresholds and force requirements for the system. Specifically, FR2 extended to read:

FR2. The system can attach to IJF fragments *under manipulation forces of 150* *N.*

while NFR3 extended to read:

NFR3. The system creates sufficient working space inside the joint *by applying forces of 300* *N.*

### Workflow, Architecture and Sub-system Design

The first step was to revisit the proposed workflow. From the various imaging and robot navigation requirements, it was inferred that we need to develop an image-guided control algorithm. For this type of activity, it is standard to use optical tracking tools and our task was to determine clinically acceptable and technically feasible points of the tool attachments.

Based on the above workflow requirements and according to the V-model of the design, the general architecture of the system was defined. Regarding the hardware architecture, based on the requirements related with the physical aspects (FR1–FR3) and space limitations (NFR12–NFR15), a modular approach was selected over a large monolithic mechanism. The testing of the entire system was performed on synthetic bones in laboratory setting and the adopted precision metric was the positional accuracy of the reduction, e.g. the normal distance between the fracture lines. In these verification tests, the entire architecture and workflow was shown to operate. Specifically, the system achieved virtual reduction of the fracture with a maximum residual positioning error of 0.95 ± 0.3 mm (translational) and 1.4° ± 0.5° (rotational) and correspondent physical reductions with an accuracy of 1.03 ± 0.2 mm and 1.56° ± 0.1°.^11^

#### Sub-System Design

*FR1* For the multi-orientation approach to fracture fragments, a hybrid geometry for the system has been designed, in the form of a serial robotic mechanism called the carrier platform (CP) for gross positioning and orientation in respect to the patient’s limb and a hexapod parallel mechanism called the robot fracture manipulator (RFM) for fine manipulation of the fragments. The CP consists of two linear and two revolute joints in a configuration that allows move around the limb of the patients and the approach from various angles. The RFM is attached to the CP in this hybrid configuration.

*FR2/NFR1/NFR2/NFR17* In order to allow the secure attachment of the system to the fragments and to cause a minimum possible damage to the surrounding soft-tissue, a new percutaneous fragment manipulation device (PFMD) that replaces traditional manipulation pins has been developed to satisfy one of the essential safety requirements. The PFMD provides the attachment of the RFMs to the bone fragments via a single incision less than 10 mm. The PFMD can be anchored to the bone mono-cortically by using a unique geometry pin (UGP), an anchoring system (AS), and a gripping system (GS). The PFMD has been characterised and its deformation is evaluated showing that for forces of 150 ± 15 N, the maximum deformations of the device is 5.8 mm.

*FR3* The fragment manipulation is achieved by the combined operation of the RFM and the Image-based navigation system. Using the data from the optical tool the system controls the motion of the RFM^8^ with system’s positioning accuracy and repeatability showing a maximum positioning RMSE of 1.18 ± 1.14 mm (translations) and 1.85 ± 1.54° (rotations). More details of the navigation control of the RFM can be found in Supplement S3.

*FR3/NFR3* DFF requires traction of the tibia to restore the original length and rotation of the joint. In the current clinical practice, this is performed by pulling the patient’s foot manually or using a traction table. This allows the surgeon to apply a constant and adjustable traction force to facilitate the reduction process.^19^ We introduced in the RAFS system a computer-controlled version of the traction table, i.e. the automated traction table (ATT).

*FR4/*NFR5 The system is semi-automated, so that the surgeon first pre-plans the reduction of the fracture in the workstation, and then the robotic system—connected to the fracture—executes the physical reduction accordingly. Moreover, the surgeon can adjust the plan intra-operatively based on the progress of the operation.^13^ For these to be achieved the host PC runs the reduction software’s graphical user interface (GUI) that creates the link between the surgeon and the robotic system. The GUI allows the surgeon to interact with CT-generated 3D models of the fracture. Virtual paths of the 3D fragment models generate corresponding motion of the robotic system.

*FR4/*NFR6/NFR7 The GUI provides the surgeon with both 2D views of each anatomical plane (i.e. sagittal, frontal, transverse) and a 3D view of CT-generated fracture models. The user controller chosen for this application is the Leap Motion, which is able to track and synthetize a 3D position and orientation (6DoF) of the hands in its workspace. Also, three foot pedals that provide on–off inputs to the system are included (1) to grab and release the fragment models, (2) to select a specific anatomical plane for interaction, and (3) to merge two fragments together that are further manipulated as one fragment.^11^

*FR5/NFR8/NFR9* A pre-operative CT scan of the fracture is taken, and the resulting dataset segmented to generate 3D models (CAD model) of each bone fragment. The models are imported and displayed in the reduction software so that the surgeon can interact with them using the GUI as described above.^11^

*FR5/NFR10* The surgeon virtually reduces the fracture using the GUI by manipulating and matching the broken fragment to move them to the original unbroken position. This generates the desired final poses for each fragment. Pre-operative planning data are stored in the system and used for intra-operative robot motion calculations to achieve the physical reduction of the fracture.^13^

*FR5/NFR11* The system is equipped with an optical tracking system (Polaris Spectra, NDI Inc., tracking accuracy 0.25 mm) which provides intra-operative real-time update of the 3D models through the optical tools attached to the orthopaedic pins inserted into the bone fragments.

**Figure 4:**
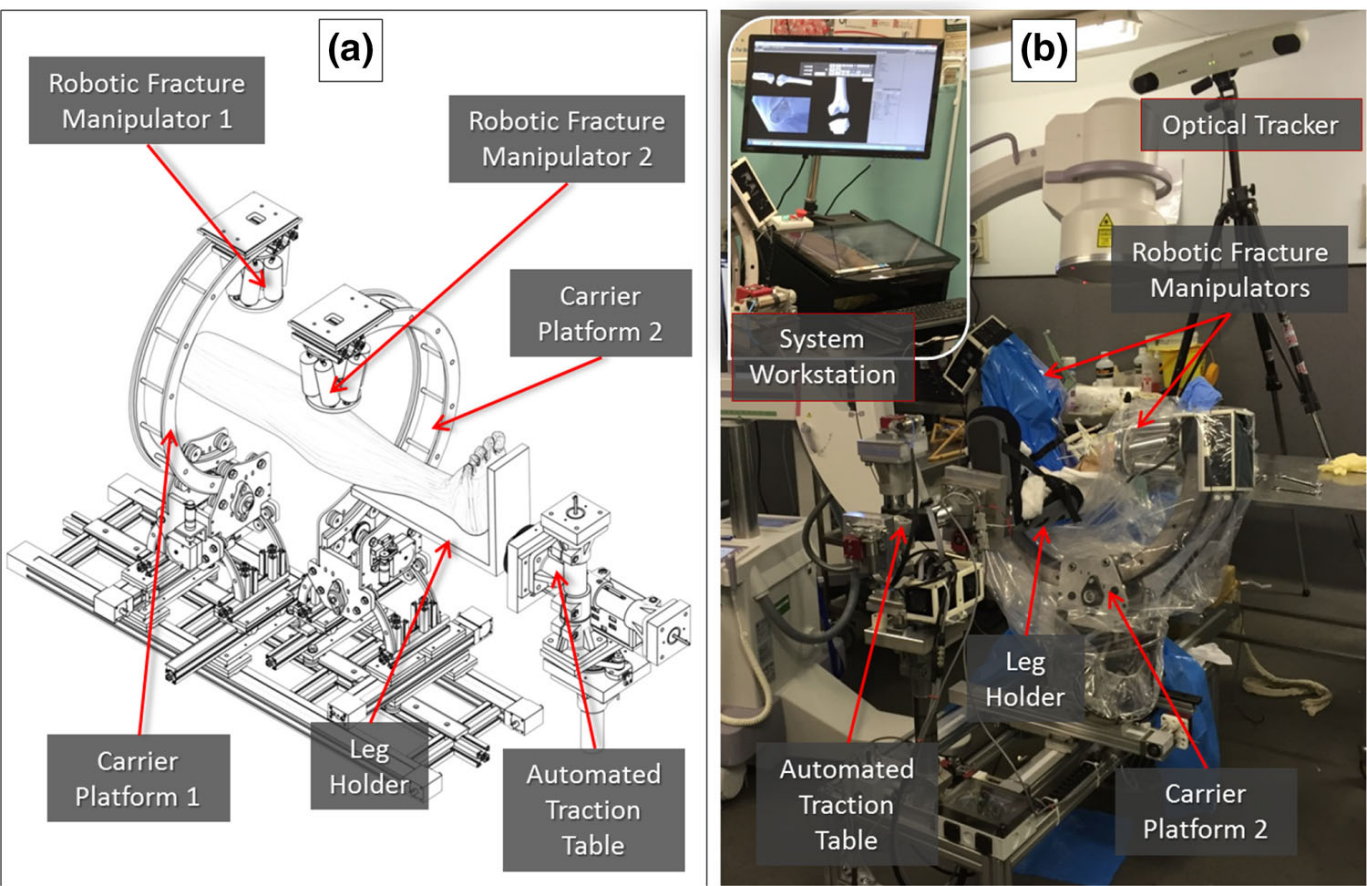
The physical parts of the RAFS system. (a) The 3D rendering of the sub-systems while (b) is the real configuration as used in the validation cadaveric trial. The optical tracker and the Image intensifier can be seen in, and in the insert the System Workstation is depicted.

*NFR4/NFR12–NFR15* These non-functional requirements are related to the geometry of the system and the way it integrates with the staff and the existing equipment in operating theatres (OR) and current practice in orthopedic surgery. To this end, the overall geometry and physical footprint of the system were considered which inspired the modular structure of the system shown Fig. 4. The different components of the system are rigidly connected, i.e. the CPs and the ATT are secured on a portable rigid wheeled frame which can be easily moved and replaced by a OR trolley.

*NFR16* To ensure the safety of the system, the latest regulations and certifications were followed in the design and development of all sub-systems. Table 4 summarises the different standards used. Special attention was given to activities that emulate a quality management system (QMS) leading to conformity to ISO13485. To this end, we focused on the design and development inputs, verification and validation, and used relevant standards as inputs to the process. Moreover, the validation test (cadaveric study) was documented according to ISO13485 regarding the acceptance criteria and statistical techniques used.

**Table 4:** Safety standards applied to the RAFS system.

Standard	Description
IEC 60601-1	Medical electrical equipment—all parts
IEC 60601-1-10:2007	Part 1–10: collateral standard: requirements for the development of physiologic closed-loop controllers
UL2601	Medical electrical equipment: general requirements for safety
IEC 60364-4-41	Low-voltage electrical installations—part 4–41: protection for safety—protection against electric shock
IEC 62304	Medical device software—software life cycle processes
IEC 60417	Power switch markings
NEMA DICOM	Digital imaging and communications in medicine
ISO/IEC 10918 ISO/IEC 14495 ISO/IEC 15444 ISO/IEC 13818	Information technology—digital compression and coding of continuous-tone still images: requirements and guidelines (JPEG)
BS EN ISO 13850 BS EN ISO 13849	Robotics safety and emergency stops
ISO 11898	Controller area network (CAN)—all parts
EN 50325-4	Industrial communications subsystem based on ISO 11898 (CAN) for controller-device interfaces—part 4: CANopen
EN 50325-5	Industrial communications subsystem based on ISO 11898 (CAN) for controller-device interfaces—part 5: Functional safety communication based on EN 50325-4
73/23/EEC 2006/95/EC	Low voltage legislation: low voltage directive (LVD)
UL E29179	Connectors for use in data, signal, control and power applications
T-REC-V.11	Electrical characteristics for balanced double-current interchange circuits operating at data signalling rates up to 10 Mbit/s (RS-422)
2002/95/EU	CAT5e
IEEE 802.3-2002	IEEE standard for information technology—local and metropolitan area networks—specific requirements—part 3: carrier sense multiple access with collision detection (CSMA/CD) access method and physical layer specifications
ISO 14971	Medical devices—application of risk management to medical devices
ISO 5725-1	Accuracy (trueness and precision) of measurement methods and results—part 1: general principles and definitions
ISO 13485	Medical devices—quality management systems—Requirements for regulatory purposes

### Validation Testing

Based on the operational and safety requirements, the most suitable validation test was the use of human cadavers (trials approved by the National Research Ethics Committee, REC Reference: 15/WM/0038, UK). The specimens used were right and left lower limbs from male (*n* = 4) and female (*n* = 3) cadavers with no bone defects on which the desired fractures were created. Specimens were collected from the proximal femur to the end of the foot. For the creation of appropriate fracture shapes (T and Y, 33-C1^3^) in a predictable and reproducible manner, an accepted technique of osteotomy was used. From the validation testing it has been shown that the system performs within the required operational requirements and achieves reductions of ≈ 1 mm and ≈ 5°.^13^

### Final Interview Study

The final interview study identified three key findings related to the process described above. Firstly, the clinical workflow presented received an average score of 3.8 out of 6, where 0 indicates *“Not at all acceptable”* and 6 *“Highly acceptable”*. With the manual actions of the surgeon, i.e. the pre-operative *Virtual Reduction* and the intra-operative actions of *Robot*–*Patient connection* and *Insertion of orthopaedic pins*, scoring 2.5 out of 6, where 0 indicates “not at all challenging” and 6 “Highly Challenging”. Secondly, regarding the optimal representation of the fracture, 17 out of 18 participants preferred a combination of 2D and 3D views (the outlier preferred 3D views only). Finally, regarding the physical dimensions of the system, 8 out of 18 preferred the current size, 8 out of 18 preferred a smaller size, and 2 out of 18 a larger size.

## Discussion

The *requirement elicitation study* provided critical insights into the difficulties and issues related with the DFF reduction. One of the most notable problems was the limited visualisation provided by the available intra-operative imaging technologies for the adoption of minimally invasive management of fractures. Moreover, the typical radiological assessment of the fracture, either with plain X-rays (pre- and Intra-operatively) or with CT scanning (pre-operatively) does not provide any information about the soft tissue damage and location. Assessment of the reduction accuracy is not in the regular practice and misalignments are often detected when follow-up morbidities occur, e.g. arthritis. Also the mind-to-hand coordination of the surgeon, due to poor visualisation renders minimally invasive procedures challenging. This prompted the development of 3D real-time image guidance for RAFS.

The second key issue that emerged from the interviews was related to the congested nature of the operation, i.e. multiple anatomical structures in a cluttered environment. This limitation is contributed primarily to the neurovascular structures and major ligament structures, especially in posterior condyle cases. The soft tissue poses a challenge in the fracture reduction both intra-operatively and post-operatively. In the first case, soft tissue can affect the quality of fracture reduction, and disrupt the correct anatomical position of the ligaments generating tension in the fragments, or tissue swelling. At the same time the soft tissue damage due to the operation, must be kept to a minimum to avoid tissue scarring and fibrosis affecting negatively the healing process. The space constraints and soft tissue constraints make not only the reduction process difficult but also keeping the fragments in place before and during fixation. Moreover, the correct fixation implant selection and positioning proves difficult both due to space constrains and pre-operative visualisation, affecting the correct anatomical restoration of the articular surface leading to post-operative arthritis.

Finally, the reduction can be impaired by the bone quality, e.g. by osteoporotic bones; both in terms of reduction and fixation. The ‘softer’ bones are prone to breaking and are more difficult to grasp and manipulate.

Based on these discussions, the first point that this investigation had to tackle was the *workflow* of the proposed intervention, specific with regards to image-guidance. In current practice there are no provisions for imaging and navigation and a new workflow was proposed where pins would be placed prior to initial CT imaging.^11^ On a second iteration of assessing the system it was found that the proposed workflow could potentially violate other requirements (e.g. NFR17) and an alternative workflow was proposed along with a technical requirement, i.e. the use of image registration prior to operation and using CT-scan data and fiducials in the theatre. The revised workflow can be seen in Fig. 3b with details of its implementation presented in Ref. 14. The workflow assessment in the final interview study was judged as acceptable by the clinicians.

The second point this investigation has achieved is the *architecture* that is fit for purpose and adaptable to the wide spectre of requirements and constrains. The three physical sub-systems were identified to be the Robot Fracture manipulator (RFM), the carrier platform (CP), and the automated traction table (ATT). For the software, sub-systems of the functional entities were identified as graphical user interface (GUI), imaging and registration (IR), navigation and high level-control (NHLC), and low-level control. The first two are implemented on a workstation and the latter two on a dedicated embedded controller.

During the design and development of the system, each *requirement* has been analysed and the final sub-systems were aligned to satisfy all of them. The main focus was on functional and non-functional requirements with each subsystem tackling a number of different requirements.

The *CP* is tackling requirements related to the wide work envelope of the system. For FR1, the two linear joints allow motion along the axis and radially around the limb, while the revolute joints allow for rotation in the perimeter of the limb and at the angles oblique to the axis of the limb.^12^ Also for NFR4/NFR12 the CPs are of such a size that allow the approach from different angles while at the same time will (1) allow space for the placement of an image-intensifier while the system is attached to the fragments Fig. 4, and (2) leave most of the surgical field free for the surgeon to manually fixate the fracture. The size of the system was also addressed in the final interview study and the clinicians were split between the current and a smaller size indicating that further investigations are needed. The detailed operation and axis of motion of the CPs and its kinematic analysis are reported in Ref. 10 and in Supplement S1. The operation of the CP is tested and verified against the set criteria.

For dealing with the key manipulation requirement, FR3, the *RFM* has been proposed. Also the compact nature of the RFM can tackle NFR15 to allow access to the surgical field. The RFM is an automated computer-controlled parallel-robot^8^ with 6 degrees-of-freedom (DOF), i.e. three translations and three rotations along/around *x*, *y*, *z* axes. The use of a parallel-robot is a preferred choice for orthopaedic applications where high load carrying capacity and precise positioning accuracy-repeatability are of paramount importance. The parallel-robot has been designed and manufactured in-house ad hoc with the desired characteristics. The struts have been developed as linear actuators based on a ball screws and a brushed DC motor with integrated gearbox and rotational encoder (RE10–MR–GP10K, Maxon Motor) providing high-torque, precise positioning (0.485 µm resolution). The 6 linear actuators produce a resulting load capacity of 360 N (force) and 12 Nm (torque) in the testing and verification process reported in Ref. 8.

For providing traction (FR3/NFR3) the ATT is proposed. The ATT is a 4-DOF mechanism (two prismatic and two revolute joints) Fig. 4, connected to the tibia through an orthopedic boot and a leg holder. The ATT allows for precise traction that will create space for the performance of reduction maneuvers. Details of the use, and testing and verification of the ATT can be found in Ref. 13 and in Supplement S1.

Addressing the issues related with anchoring the system on the bone *a new PFMD* was designed and tested composed of three elements the UGP, the GS, and the AS. The UGP is a custom-designed non-cannulated 6 mm diameter orthopaedic manipulation pin with 4 distinctive cross-sections. These sections allow for the different functionalities, i.e. connection to RFM via the GS, attachment of an optical tool for navigation purposes,^10^,^11^ attachment to the AS, and a threaded metric M6 section that is screwed into a single cortical plane of the fragment exhibiting good pull-out characteristics. The AS (Fig. 2b) is a custom designed system that firmly embeds the UGP into the bone fragment using a drilling template (DT) to hold four stainless steel nails that the surgeon drills into the bone fragment. More details about the testing and verification of the PFMD can be found in Supplement S2.

The *Navigation and Tracking System* is based on the Polaris optical tracker. The tracking device is using optical tools that are being placed on crucial parts of the system, namely the fragments, the RFM, and the tibia in the case of DFF. To enable intra-operative image guidance, the relative position of each pin with respect to the bone fragment in which it is inserted is calculated through intra-operative surgical registration.^14^ Once the relative pose of each pin bone is known, and assuming that it does not change over time (i.e. the object constituted by the pin and the bone fragment is considered rigid), the pose of each bone fragment is updated in real-time using the optical tracker by connecting an optical tool to the pin.^13^ This depicts the actual pose of each fragment in the 3D space during the surgery. Intra-operative imaging allows surgeon to monitor progress of the physical fracture reduction performed by the robotic system. More information about the testing of the navigation and tracking system can be found in Refs. 13, 14 and Supplement S3.

**Figure 5:**
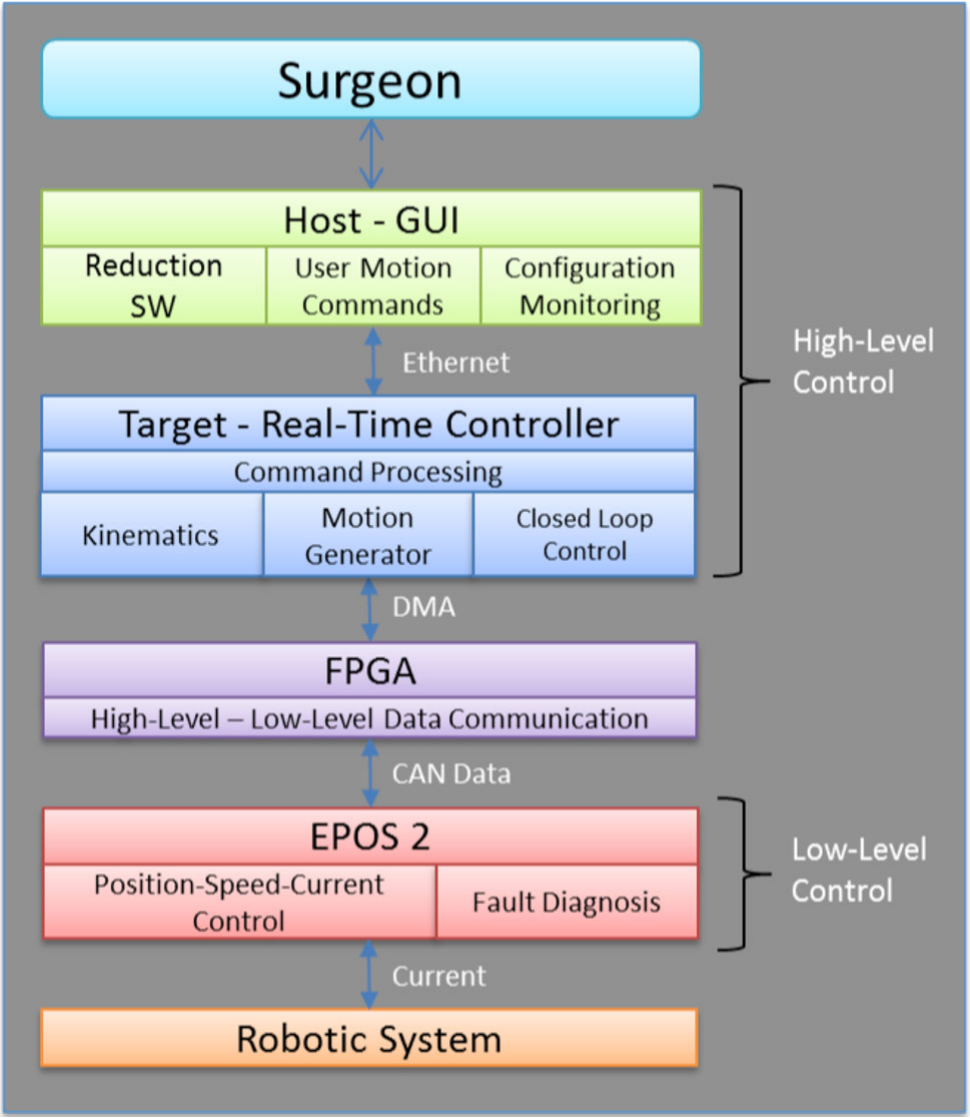
The software and information sub-systems architecture. The place of the surgeon as being always in control can be seen here. There are two levels of control sub-systems the high-level ones that are dealing with the complex decisions and the FPGA (field programmable logic array) low-level control that are implementing the required action by the robotic mechanisms.

The tracking information is used for the *Control* of the system and Fig. 5 shows the control architecture of the system, with the surgeon in control of the robotic device and planning the surgical procedure from a workstation. The system employs a host–target structure composed by a PC (host) and a real-time controller with FPGA (target), and a low-level motor controller. The target controller (compactRIO-9068, National Instruments) processes the surgeon’s virtual reduction, and generates motion commands which are sent to the low-level motor controller (EPOS2 24/3, Maxon Motor) that executes the movement of the robotic system to achieve the physical reduction of the fracture.^11^ More details about the testing of the control scheme can be found in Supplement S3.

The interaction with the user is ensured via the specially design *GUI* in the workstation of the system. The 2D views (projections) of the fracture allow the surgeon to perform the virtual reduction. The 3D view allows the surgeon to move the camera around the model in the virtual environment to assess the outcome of the reduction. The use of 2D and 3D views was favoured by the clinicians as indicated in the final interview study. The surgeon intuitively interacts with the 3D models using their hands through a user controller to virtually reduce the fracture in the virtual environment. This way the requirements for pre-operative planning but also intra-operative control of the process, i.e. under sterile conditions can be achieved.

## Conclusions

This paper presented a user-centred approach for the design and development of a novel medical device. The interviews with the surgeon at an early stage of the medical device development allowed the research team to capture the needs and current issues of the clinical practice. Following a design and development approach based on established methods like the V-model of design the final system has been built and tested to perform within the requirements. The final results demonstrated that appropriate design methods allow the development of a complex system within time frames and constrains to achieve its goals. Future works include the formulation of a design and development approach which can be applicable to other healthcare systems requiring the input from the users.

## Electronic supplementary material

Below is the link to the electronic supplementary material.

Supplementary material 1 (PDF 1647 kb)
