# Supplementary material for: Robot-Assisted Fracture Surgery: Surgical Requirements and System Design
Source: Ann Biomed Eng. 2018 Mar 9;46(10):1637–49. doi: 10.1007/s10439-018-2005-y (PMC6153987; doi:10.1007/s10439-018-2005-y)
Supplement: Supplementary file 1 — Supplementary material 1 (PDF 1647 kb) [file 10439_2018_2005_MOESM1_ESM.pdf]

Supplement S1

Carrier Platform (CP) Kinematics

The forward kinematics of the CP is based on the Denavit–Hartenberg (DH) analysis (Fig.S0.A). The DH parameters are given based on the joint vector:

$$\boldsymbol{q} = [L_z\ L_y\ T\ R] \qquad \text{(A0.1)}$$

where, L_z is the linear motion along the axis of the limb, L_y is the linear motion perpendicular to L_z, T is the tilting revolute joint around L_y, and R is the revolute rotation around L_z, as shown Fig.S0.A.

The transformation matrix between the CP origin (O) and the RFM origin (O_{RFM}) is given by:

$$^{\boldsymbol{O}}\boldsymbol{T}_{\boldsymbol{O_{RFM}}} = \begin{bmatrix} {}^{O}R_{O_{RFM}} & {}^{O}P_{O_{RFM}} \\ 0\quad 0\quad 0 & 1 \end{bmatrix} =$$

$$= \begin{bmatrix} -s_T & -c_T s_R & -c_R c_T & X_{FK} \\ 0 & c_R & -s_R & Y_{FK} \\ c_T & -s_R s_T & -c_R s_T & Z_{FK} \\ 0 & 0 & 0 & 1 \end{bmatrix} \qquad \text{(A0.2)}$$

$$X_{FK} = l_1 + l_2 + l_3 c_T + l_4 c_R c_T + T_A c_R c_R - T_B c_T s_R$$

$$Y_{FK} = T_B c_R - L_y + l_4 s_R + T_A s_R$$

$$Z_{FK} = L_Z + l_3 s_T + l_4 c_R s_T + T_A c_R s_T - T_B s_R s_T$$

where, c_x is *cos(x)*, s_x is *sin(x)*, T_A and T_B are the offsets along x and z axes (given by structural dimensions).

For a desired target position of the connected RFM:

$$^{\boldsymbol{RFM}}\boldsymbol{P_d} = [x_d\ y_d\ z_d\ \vartheta x_d\ \vartheta y_d \vartheta z_d] \qquad \text{(A0.3)}$$

analytical solution for the inverse kinematics can be derived by solving the forward kinematics (A0.2) to find CP parameters (refer to (A0.1) and Fig.S0.A):

$$R = \theta z_d$$

$$T = \text{acos}\ \frac{x_d - l_1 - l_2 - T_A c_R c_T}{(l_3 + l_4 c_R - T_B s_R)} \qquad \text{(A0.4)}$$

$$L_y = T_B c_R + l_4 s_R + T_A s_R - y_d$$

$$L_Z = Z_{FK} - l_3 s_T - l_4 c_R s_T - T_A c_R s_T + T_B s_R s_T$$

Robotic Fracture Manipulator (RFM) Kinematics

The RFM has a parallel-robot configuration with six struts. The inverse kinematics for the parallel-robot is derived using the loop closure approach for each of the struts (refer to Fig.S0.B). This is a vector method that gives relationships between the key points of the robot structure, i.e. O the centre of the base platform, O_{EE} the centre of the end-effector, A_i and B_i are the points where each strut i is attached to the base and the moving platform respectively. Their relation is given in (A0.5).

$$\overrightarrow{OO_{EE}} = \overrightarrow{OA_i} + \overrightarrow{A_iB_i} + \overrightarrow{B_iO_{EE}} \qquad \text{for } i=1\ldots6 \qquad \text{(A0.5)}$$

The $\overrightarrow{OO_{EE}}$ vector is the desired position of the end-effector in the reference frame of the base platform, vectors $\overrightarrow{OA_i}$ and $\overrightarrow{B_iO_{EE}}$ are known vectors based on the robot's geometry, usually the diameter of the inscribed circle for the plate and the internal angles of the hexagon. Finally, vector $\overrightarrow{A_iB_i}$ is the vector of each strut. By representing each point in its three-dimensional vector form, i.e. (x_n, y_n, z_n) we can calculate the magnitude of this vector using (A0.6).

$$\left\|\overrightarrow{A_iB_i}\right\| = \sqrt{\left(x_{B_i} - x_{A_i}\right)^2 + \left(y_{B_i} - y_{A_i}\right)^2 + \left(z_{B_i} - z_{A_i}\right)^2} \qquad \text{for } i=1\ldots6 \qquad \text{(A0.6)}$$

The length values from (A0.6) are the commands delivered to the control system that synchronises the six actuators using a velocity feed-forward control scheme. This is performed by adjusting the axis velocities based on the leading manipulator axis, i.e. the axis performing the slowest motion for a given point-to-point motion step. The velocity profile for each axis is calculated using the following equation:

$$\dot{\vartheta}_i = \frac{\ddot{\vartheta}_{MAX} \cdot t_e}{2} - \sqrt{\left(\frac{\ddot{\vartheta}^2_{MAX} \cdot t_e^2}{4}\right) - \left(s_{i,e} \cdot \ddot{\vartheta}^2_{MAX}\right)} \qquad \text{for } i=1\ldots6 \qquad \text{(A0.7)}$$

where:

$$t_e = \frac{s_e}{\dot{\vartheta}_{MAX}} + \frac{\dot{\vartheta}_{MAX}}{\ddot{\vartheta}_{MAX}} \qquad \text{(A0.8)}$$

t_e is the end time of the trapezoidal velocity profile of the leading robot axis; s_e is the maximum axis displacement.

This synchronisation algorithm is essential to simultaneously control all parallel-robot actuators and to ensure that all actuators start and stop at the same time.

Automated Traction Table (RFM) Kinematics

The forward kinematics of the ATT is based on the Denavit-Hartenberg (DH) analysis (Fig.3). The DH parameters are defined using the joint vector:

$$\boldsymbol{q} = [\theta_1 \; d_2 \; d_3 \; \theta_4] \qquad (1)$$

where, θ_1 is the rotation of the ATT around the axis perpendicular to the limb, d_2 is a displacement perpendicular to the limb axis, d_3 is a displacement along the limb axis, and θ_4 is the rotation around the limb axis. The transformation matrix between the ATT origin {*O*} and the base of the foot reference frame {O_{RFF}} is given by:

$${}^{O}\boldsymbol{T}_{O_{RFF}} = \begin{bmatrix} c_1c_4 & -c_1s_4 & -s_1 & -d_3s_1 \\ s_1c_4 & -s_1s_4 & c_1 & d_3s_1 \\ -s_4 & -c_4 & 0 & d_2 + l_1 + l_3 \\ 0 & 0 & 0 & 1 \end{bmatrix} \qquad (2)$$

where, c_x is *cos(x)* and s_x is *sin(x)*. For a desired (*d*) target position and configuration of RFF in respect to {*O*} given by the vector:

$${}^{ATT}\boldsymbol{P}_d = \left[X_d \; Y_d \; Z_d \; \theta_{X_d} \; \theta_{Y_d} \; \theta_{Z_d}\right] \qquad (3)$$

where, $X_d \; Y_d \; Z_d$ are the Cartesian coordinates in respect to {O} and $\theta_{X_d} \; \theta_{Y_d} \; \theta_{Z_d}$ the Euler angles in respect to {O}, an analytical solution for the inverse kinematics of the ATT to provide the desired joint parameters is given by:

$$\begin{gathered} \theta_1 = atan2(X_d, Y_d) \\ \theta_4 = \theta_{Y_d} \\ d_2 = Z_d - (l_1 - l_3) \\ d_3 = \sqrt{X_d^2 + Y_d^2} \end{gathered} \qquad (4)$$

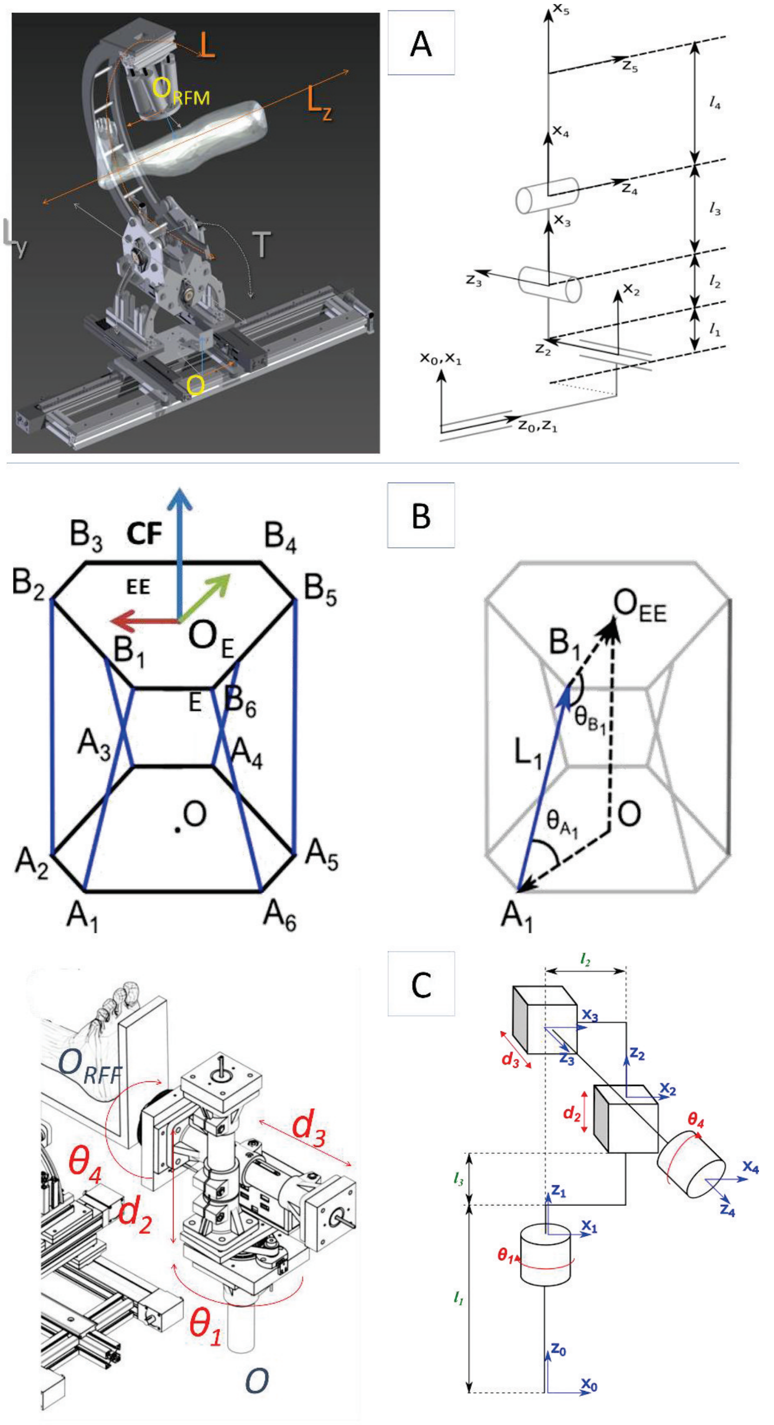

FIGURE S1. Kinematic chain for the Carrier Platform (A), for the Robotic Fracture Manipulator (B), and for the Automated Traction Table (C). Figures from [43] and [37].

Supplement S2

This is part of work under review[29]

Percutaneous Fragment Manipulation Device (PFMD)

The PFMD consists of the Unique Geometry Pin (UGP), the Anchoring System (AS), and the Gripping System (GS) (FigureS2).The UGP (FigureS2A) is a custom-designed non-cannulated orthopaedic manipulation pin (6mm diameter (D), 142mm length (L)). It has 4 distinctive cross-sections: *(i) gripping section* (L=12mm) a cylindrical section of 4mm diameter, to be connected to the GS; *(ii) tool section* (L=33mm), a three-flat-faces unique geometry to which a tool (e.g. optical tool for real time tracking) can be mounted in a unique orientation, enabling the 3D imaging system; *(iii) anchoring system section* (L=67mm), a two-flat-faces geometry on which the AS is fixed, because of the flat surfaces no rotation around the axis of the UGP will be possible; *(iv) threaded section* (L=30mm), an M6 metric thread (maj. diam. 5.91mm, min. diam. 4.74mm, pitch 1mm), screwed into a single cortical plane of the fragment. The specified pitch, diameter and number of turns of the thread were selected optimising manufacturing limitations and pull-out characteristics[S2-1]. Deformations of the UGP via the tool section are recorded using a commercial optical tracking system (Polaris Spectra, NDI Inc., tracking accuracy 0.25mm).

The AS (FigureS2B_1) is a custom designed system that firmly connects the UGP with a bone fragment using a holding ring called a Drilling Template (DT) and three or four 2mm (diameter) stainless steel K-wires. The DT (FigureS2B_2) has a total of 5 openings, one central with two-flat-faces for the UGP, and four circular ones for the K-wires. The surgeon drills the UGP into the bone fragment following the normal procedure for pin placement, then slides the DT into the *anchoring system section* and drills the 4 K-wires into the bone fragment through the holes on the DT. The K-wires cross through the holes of the DT and into the bone firmly stabilising the UGP to the fragment.

The GS (FigureS2C) is mounted on the RFM end-effector and consists of an adjustable spherical joint that can freely orient a specially designed insert which fits in the *gripping section* of the UGP. This configuration ensures that the force/torque applied by the RFM is fully transferred to the bone fragment to achieve the desired anatomical reduction.

The PFMD was tested in Finite Element Analysis (FEA) simulation, ex-vivo animal tests, and a cadaveric trial to ensure that when under high manipulation forces does not deform being the plastic region, i.e. breakage point. The results were consistent between the different trials and are given in Table S2. Non breakage was recorded in any of the tests conducted.

Table S2, Deformation Data for PFMD reported in RMD

**Loading**	**FEA Simulation**	**Ex-vivo Animal**	**Cadaveric Trial**
80N±8N	2.8mm	2.97mm	3.06mm
150±15N	5.8mm	4.44mm	5.19mm

RMD = resultant maximum displacement (translational and rotational)

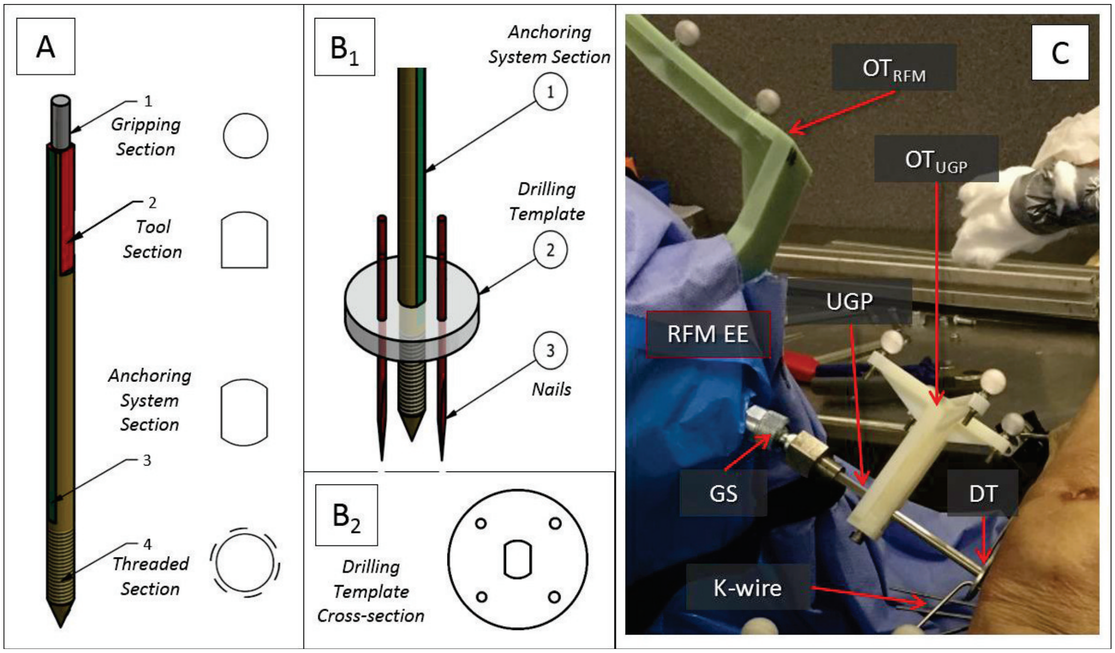

Figure S3: Robot-Bone Fixation System. CAD drawings of the Unique Geometry Pin (UGP) and its different cross-sections (A), he anchoring system (AS) (B1) and a detail of the Drilling Template (DT) (B2). The UGP is secured in the Gripping System (GS) and connects the RFM end-effector with the bone fragment. Optical tools are placed on the UGP (OTUGP) and the RFM (OTRFM) allowing the measurement of their relative pose (C).

S2-1 Chapman, J. R. *et al.* Factors affecting the pullout strength of cancellous bone screws. *Trans. Soc. Mech. Eng. J. Biomech. Eng.* **118,** 391–398 (1996).

Supplement S3

RAFS System Control Strategy

The RAFS system architecture, employs a host-target structure based on a PC (host) running a graphical user interface (GUI) and a real-time controller with FPGA (target, NI CompactRIO 9068, National Instruments) running the high-level control[7,10]. Surgeon's virtual reduction (VR) input is processed by the high-level controller generating motion commands for the low-level controller (EPOS 2 24/3 (special edition), Maxon Motor) which, consequently, performs physical fracture reduction. The control strategy is based on a combination of open-loop and closed-loop position control. Open-loop position control of the ATT takes signals from four motor encoders to provide the required pose of the ATT in the task space. Visual feedback provided by the optical tracker facilitates a closed-loop vision-based position control on CPs and RFMs by placing optical tools on UGPs and the end-effectors[7,10]. This is necessary to guarantee the required reduction accuracy.

The RAFS system control strategy is summarized in Fig.S1. The open-loop control of the ATT is based on the kinematics reported in the manuscript. The surgeon defines the desired pose for the ATT ${}^{ATT}\boldsymbol{P}_d$ through the GUI by checking the actual pose of the fragments using the navigation system. The desired position of the ATT is defined by solving the kinematics. The system calculates the motion commands for the motors to reach the desired pose ${}^{ATT}\boldsymbol{P}_d$.

Vision-based position control of the RAFS system is based on the kinematics of the CPs and RFMs. Considering the clinical workflow, firstly the CPs position the RFMs close to the UGPs allowing the surgeon's assistant to connect the RFMs end-effectors to the UGPs. The actual poses of the UGPs are provided by their optical tools (OT_{F1}, OT_{F2}) and represent the desired staring (*ds*) poses for the RFMs, i.e. ${}^{RFM1}\boldsymbol{P}_{ds} = {}^{OTF1}\boldsymbol{P}_a$ and ${}^{RFM2}\boldsymbol{P}_{ds} = {}^{OTF2}\boldsymbol{P}_a$. The system calculates the required movement for CPs in order to position RFM1 in ${}^{RFM1}\boldsymbol{P}_{ds}$ and RFM2 in ${}^{RFM2}\boldsymbol{P}_{ds}$, by solving the kinematics for CP1 and CP2, respectively as described in [7].

Once the RFMs and the UGPs are connected, the surgeon defines the desired trajectories for F1 and F2 (${}^{F1}\boldsymbol{Tj}_d$, ${}^{F2}\boldsymbol{Tj}_d$) by virtually reducing the fracture. Trajectories ${}^{F1}\boldsymbol{Tj}_d$ and ${}^{F2}\boldsymbol{Tj}_d$ are defined as a number of desired intermediate (*di*) poses for F1 and F2 (${}^{F1}\boldsymbol{P}_{di}$, ${}^{F2}\boldsymbol{P}_{di}$) between their initial (${}^{F1}\boldsymbol{P}_s$, ${}^{F2}\boldsymbol{P}_s$) and final (${}^{F1}\boldsymbol{P}_d$, ${}^{F2}\boldsymbol{P}_d$) poses.

$$
\begin{aligned}
{}^{F1}\boldsymbol{Tj}_d &= [\ {}^{F1}\boldsymbol{P}_s, \dots, {}^{F1}\boldsymbol{P}_{di}, \dots, {}^{F1}\boldsymbol{P}_d\ ] \\
{}^{F2}\boldsymbol{Tj}_d &= [\ {}^{F2}\boldsymbol{P}_s, \dots, {}^{F2}\boldsymbol{P}_{di}, \dots, {}^{F2}\boldsymbol{P}_d\ ]
\end{aligned}
\qquad (A1.1)
$$

For each $^{F1}\boldsymbol{P}_{di} \in {}^{F1}\boldsymbol{Tj}_{d}$ and $^{F2}\boldsymbol{P}_{di} \in {}^{F2}\boldsymbol{Tj}_{d}$, the corresponding desired poses in the task space for the RFMs $^{RFM1}\boldsymbol{P}_{di} \in {}^{RFM1}\boldsymbol{Tj}_{d}$ and $^{RFM2}\boldsymbol{P}_{di} \in {}^{RFM2}\boldsymbol{Tj}_{d}$ are calculated (see Supplemental S2) to achieve the fracture reduction.

During the physical reduction, the actual pose of RFMs $^{RFM1}\boldsymbol{P}_{a}$ and $^{RFM2}\boldsymbol{P}_{a}$ provided by the optical tracker (through OT_{RFM1} and OT_{RFM2}) is compared to the desired intermediate poses $^{RFM1}\boldsymbol{P}_{di}$ and $^{RFM2}\boldsymbol{P}_{di}$ generating the pose errors $^{RFM1}\boldsymbol{E_P} = (\boldsymbol{E_{T1}}, \boldsymbol{E_{R1}})$ and $^{RFM2}\boldsymbol{E_P} = (\boldsymbol{E_{T2}}, \boldsymbol{E_{R2}})$. $\boldsymbol{E_{T1}}$ and $\boldsymbol{E_{T2}}$ are the translational position errors expressed as the difference between the desired and actual for each axis, while $\boldsymbol{E_{R1}}$ and $\boldsymbol{E_{R2}}$ are the rotational errors calculated using the quaternion errors between the desired and actual orientations for each axis. Calculation methodology for $\boldsymbol{E_P}$ is fully reported in [7]. At each processing time step (n), twelve PI (proportional and integral) controllers, one for each translational ($x_1, y_1, z_1, x_2, y_2, z_2$) and rotational ($\vartheta x_1, \vartheta y_1, \vartheta z_1, \vartheta x_2, \vartheta y_2, \vartheta z_2,$) axis of each RFM, generates motion commands $^{RFM1}\boldsymbol{\varphi}(n)$ and $^{RFM2}\boldsymbol{\varphi}(n)$ to adjust the pose of the end-effectors in the task space, by minimizing $^{RFM1}\boldsymbol{E_P}$ and $^{RFM2}\boldsymbol{E_P}$. The control laws and the PI tuning parameters are reported in [7]. Force and torque data provided by the load cells mounted on the ATT and the RFMs end-effectors, namely $\boldsymbol{F_a} = ({}^{ATT}\boldsymbol{F_a}, {}^{RFM1}\boldsymbol{F_a}, {}^{RFM2}\boldsymbol{F_a})$, are used as a safety feature to stop the system if predefined thresholds $\boldsymbol{F}_{th} = ({}^{ATT}\boldsymbol{F}_{th}, {}^{RFM1}\boldsymbol{F}_{th}, {}^{RFM2}\boldsymbol{F}_{th})$ are exceeded, avoiding damage to the patient[7].

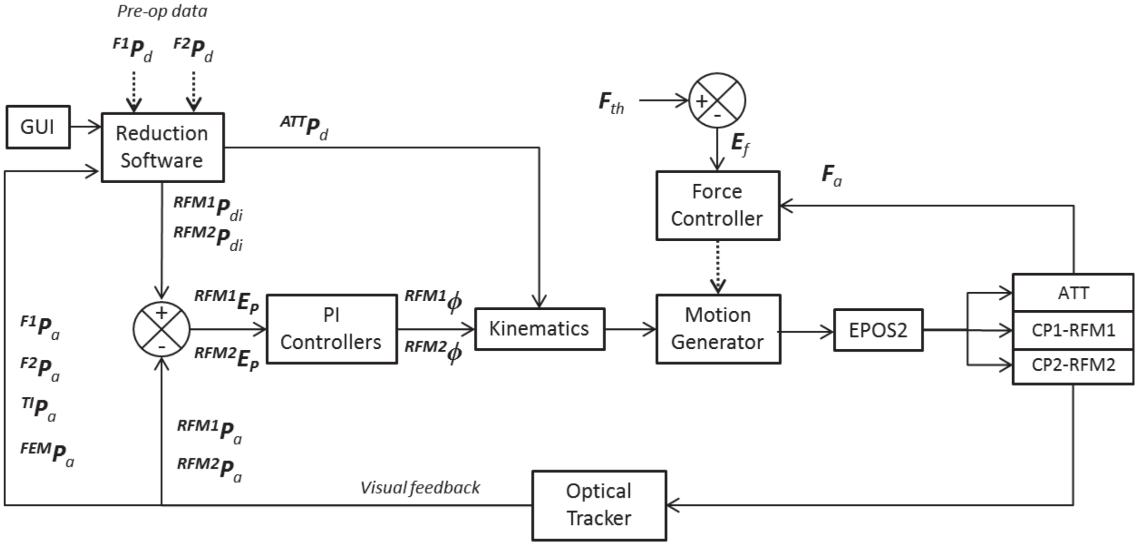

FIGURE S3. RAFS system control circuit.
